# Age-Related Decline in Myelin Markers and Oligodendrocyte Density in Rhesus Macaque Prefrontal Cortex

**DOI:** 10.1523/ENEURO.0418-25.2026

**Published:** 2026-04-14

**Authors:** Ying Zhang, Li Yao, Longbao Lv, Baizhen Zhao, Kai Zhong, Jiali Li

**Affiliations:** ^1^Key Laboratory of Genetic Evolution and Animal Models, Key Laboratory of Animal Models and Human Disease Mechanisms of Yunnan Province, National Research Facility for Phenotypic and Genetic Analysis of Model Animals (Primate Facility), National Resource Center for Non-Human Primates, Kunming Institute of Zoology, Chinese Academy of Sciences, Kunming, Yunnan 650201, China; ^2^Hackensack Meridian Health Center for Discovery and Innovation, Nutley, New Jersey 07110; ^3^High Magnetic Field Laboratory, Chinese Academy of Sciences, Hefei, Anhui 230031, China; ^4^JFK Neuroscience Institute, Hackensack Meridian Health JFK University Medical Center, Edison, New Jersey 08820; ^5^Department of Neurology, Hackensack Meridian School of Medicine, Nutley, New Jersey 07110

**Keywords:** brain aging, myelin, oligodendrocyte precursor cell, oligodendrocyte, rhesus macaque

## Abstract

Age-related alterations in myelin are a prominent feature of brain aging, yet how myelin-associated markers and oligodendrocyte lineage cell populations change across the primate lifespan remains incompletely characterized. Here, we provide a multimodal, cross-sectional analysis of myelin-related imaging and cellular markers in the prefrontal cortex (PFC) of age-matched both male and female rhesus macaques across postnatal development and aging using a multimodal approach combining magnetic resonance imaging (MRI), histological analysis, immunohistochemistry, and RNAscope in situ hybridization. We quantified regional gray and white matter volumes and myelin water fraction measures in prefrontal cortex (PFC) subregions BA9 and BA46 across four age groups: 5, 10, 15, and 30 years. Myelin water fraction and regional brain volumes exhibited age-dependent increases from childhood through adolescence, peaking at 15 years, followed by a decline in aged animals. Histological analyses revealed age-associated changes in myelin organization and the presence of myelin fragments within Iba1-positive microglia, along with dynamic alterations in the density of cells expressing oligodendrocyte lineage-associated markers, including Olig2 and oligodendrocyte precursor cell (OPC)-associated markers, in BA9 and BA46. OPC density displayed a nonlinear, age-associated pattern across developmental and aging stages, coinciding temporally with changes in myelin-associated imaging measures. Our findings define an age-related framework of myelin alterations and oligodendrocyte lineage markers in the primate prefrontal cortex. This work establishes a reference dataset for oligodendrocyte lineage dynamics across the lifespan in a translationally relevant primate model, providing a foundation for future mechanistic and interventional studies of myelin maintenance during brain aging.

## Significance Statement

Myelin integrity is essential for efficient neural signaling and is known to change with age, yet detailed characterization of myelin-associated features across the primate lifespan remains limited. Using a multimodal imaging and histological approach, this study documents age-associated changes in myelin-related markers and oligodendrocyte lineage cell density in the prefrontal cortex of rhesus macaques from development through aging. The data define normative age-related patterns in myelin-associated imaging measures, microglial engagement with myelin, and oligodendrocyte lineage marker expression. This work provides an important reference for interpreting myelin alterations in aging and neurodegenerative disease and informs future studies aimed at understanding and preserving white matter health across the lifespan.

## Introduction

Brain aging involves numerous changes, including protein dysfunction, epigenetic modifications, mitochondrial decline, and disruptions in nutrient transmission pathways (Lopez-Otin et al., 2013). Among these, the degradation of myelin integrity and oligodendrocyte dysfunction has gained prominence as significant contributors to the aging brain (Peters, 2002; Bartzokis, 2004; Yeung et al., 2014; Hill et al., 2018; Nasrabady et al., 2018). Myelin, a dense, multilayered membrane produced by oligodendrocytes, wraps axons to insulate them and accelerate signal conduction (Sobottka et al., 2011; Fields, 2014; Snaidero et al., 2017). The loss of myelin integrity is observed in various conditions involving cognitive decline, such as Alzheimer's disease and schizophrenia (Edgar and Sibille, 2012; Zhang et al., 2019), and oligodendrocyte degradation, often linked to DNA damage, is known to precede amyloid pathology in Alzheimer's disease (Tse et al., 2018).

Magnetic resonance imaging (MRI) studies show that white matter volume declines significantly after age 50 (Sowell et al., 2003). Electron microscopy has further revealed that aging-related brain changes primarily involve alterations in myelinated nerve fibers rather than neuron loss (Peters, 2009). Common myelin defects include splitting of the dense myelin line, accumulation of cytoplasmic inclusions, and “balloon-like” myelin bubbles, which appear as light-microscopic holes originating from degenerative myelin structures (Feldman and Peters, 1998). Additional features, such as multilayered myelin folds, increase with age and are partially cleared by microglia, which phagocytose damaged myelin (Safaiyan et al., 2016; Hill et al., 2018). However, aging microglia show elevated lipofuscin levels, reflecting their accumulation of indigestible myelin remnants (Safaiyan et al., 2016).

Oligodendrocytes, derived from dorsal or ventral oligodendrocyte precursor cells (OPCs), autonomously myelinate distinct brain regions (Kessaris et al., 2006; Vigano et al., 2013). Disruptions in axonal surface properties, neurotransmitter release, or electrical signals within the local environment can impair oligodendrocyte function (Almeida et al., 2011). These cells rely on local signals to maintain axonal activity and integrity, which is essential for their functionality (Nave and Werner, 2014). Myelin sheaths are not static structures; they undergo continuous remodeling. In both the central nervous system (CNS) and peripheral nervous system (PNS), myelin regeneration is a slow process, with OPCs continuing to proliferate and differentiate throughout life. In healthy adult mice, myelin repair is gradual, with longer myelin segments and fewer nodes of Ranvier compared with earlier stages of life (Young et al., 2013).

After adulthood, the ratio of white matter volume to cortical volume increases, and functional activities such as reading, playing piano, or juggling can stimulate myelin formation in the human brain (Tamnes et al., 2010; Liu et al., 2012; Nave and Werner, 2014). Similarly, motor task training in adult mice enhances myelin formation in specific brain regions, while social isolation in both juvenile and adult mice negatively affects myelination (Liu et al., 2012; Sampaio-Baptista et al., 2013). Myelin formation is a complex process involving the proliferation, migration, and differentiation of OPCs into mature oligodendrocytes capable of wrapping axons to form myelin sheaths (Sampaio-Baptista and Johansen-Berg, 2017). This differentiation occurs in a stepwise manner: (1) multifunctional neural progenitor cells (NPCs) in the embryonic stage give rise to oligodendrocytes; (2) NPCs differentiate into primitive oligodendrocyte progenitor cells (pri-OPCs) (OLIG1/2+/A2B5+/PDGFRalow); (3) pri-OPCs mature into OPCs (PDGFRahigh/NG2+); (4) OPCs develop into immature oligodendrocytes (O4+/CNP+), which then transition into CC1+-positive immature oligodendrocytes; and (5) mature oligodendrocytes (MBP+/PLP+/MOG+) are formed (Gregath and Lu, 2018). Each stage is regulated by precise extracellular and intracellular signals, including transcription factors such as OLIG1/2, Sox10, Yy1, Nkx2.2, and Myrf, as well as epigenetic mechanisms that modulate chromatin states (Wegner, 2008; Zuchero and Barres, 2013; Emery and Lu, 2015).

In mice, myelin repair persists throughout life, but aging diminishes OPC numbers and reduces their differentiation capacity, impairing myelin regeneration (Goodell and Rando, 2015; Tse et al., 2018). Significant structural and functional changes also occur in the aging brain. In rhesus macaques, mature oligodendrocytes in aged brains swell along their protrusions (LeVine and Torres, 1992; Peters, 2009). Reduced myelin integrity, particularly in the prefrontal cortex, has been associated with cognitive decline in both humans and nonhuman primates (Grydeland et al., 2013; Glasser et al., 2014). However, the biological mechanisms driving these changes in myelin integrity during brain aging remain poorly understood.

In this study, we used a combination of MRI, RNAscope, histology, and immunofluorescence to investigate the relationship between aging, myelin integrity, and oligodendrocyte function in rhesus macaques. Our findings demonstrate that myelin intensity in the prefrontal cortex (PFC) increases during early life, peaks around 15 years, and subsequently declines with age. This decline is accompanied by the accumulation of myelin fragments in microglia and a decrease in MBP-positive oligodendrocytes and OPCs. These results suggest that age-related OPC loss plays a key role in the progressive loss of myelin integrity in the aging brain.

## Materials and Methods

### Ethical statement

All animal procedures were in strict accordance with the guidelines for the National Care and Use of Animals approved by the National Animal Research Authority (China) and the Institutional Animal Care and Use Committee (IACUC) of the Kunming Institute of Zoology, Chinese Academy of Sciences. The nonhuman primate cares and experimental protocols were approved by the Ethics Committee of Kunming Institute of Zoology and the Kunming Primate Research Center, Chinese Academy of Sciences (AAALAC accredited), and the methods were carried out by the approved guidelines (Approval No: SYDW-20140311).

### Animals and samples collection

Frozen postmortem tissue samples from rhesus macaque were obtained from the Kunming Primate Research Center of the Chinese Academy of Sciences (KPRC). Brain regions were systematically collected from well-characterized rhesus monkeys born and raised at the KPRC in outdoor, 6 acre enclosures that provide a naturalistic setting and normal social environment. Extensive health, family lineage, and dominance information were maintained on all animals. According to a widely used macaque brain atlas and brainmaps (HTTP://www.Brainmaps.org), tissues spanning eight anatomically distinct regions were selected and collected from each specimen. For MR scan, instant postmortem tissue samples from postnatal female rhesus monkeys were provided by Kunming Primate Research Center of the Chinese Academy of Sciences (KPRC). Thirty age- and sex-matched macaques age from 5 to 30 years old were divided into four age groups (5, 10, 15, and 30 years). Detailed information about macaques is provided in Extended Data Figure 1-1. The brains were acquired following approved protocols from the Kunming Institute of Zoology, Chinese Academy of Sciences. The whole postmortem brains were obtained at necropsy immediately following killing for non-study-related reasons. The brain was extracted from the skull and submerged in a 500 ml solution of 4% paraformaldehyde (PFA) within minutes of killing. After a fixation period of at least 4 weeks, whole postmortem brains were transferred to a 500 ml solution of phosphate-buffered saline for 1 week. The brain was positioned in an oval-shaped container filed with FOMBLIN perfluoropolyether (Solvay Specialty Polymers). The container was generated by 3D printing and was adapted to the outer surface of the brain, such that the MR signal can be maximized. The brain immersed in perfluoropolyether was vacuumed for at least 3 d under 0.1 atmosphere pressure to remove all air bubbles in the sample before MR scans. After MR data acquisition, the brains were cleaned thoroughly with saline and maintained in 4% PFA.

### MRI acquisition and image processing

MRI was performed on a 9.4 T 40 cm MRI system (Agilent). A conformal quadrature RF coil with an inner diameter of 95 mm was used to acquire all macaque images. Both aging 2D and 3D gradient echo (GE) sequences were used. For 2D GE sequence, TR/TE was 2,800/14 ms; matrix size, 384 × 384 × 100 mm^3^; FOV, 80 × 65 × 50 mm^3^; flip angle, 80. For 3D GE sequence, TR/TE was 45/11.5 ms; matrix size, 384 × 384 × 384; FOV, 80 × 65 × 50 mm^3^; flip angle, 10; and the average scan time, 1.5 h. 2D scans were repeated eight times and subsequently averaged for each macaque brain to improve the SNR. All data processing were done on a Linux computer with 8 CPU cores and 16 GB of RAM. MR image preprocessing was performed by home-built intensity bias correction software based on Matlab (The MathWorks) together with 3D Slicer (https://www.slicer.org/), CMTK (https://www.nitrc.org/projects/cmtk/), and ITK-SNAP (version 3.6.0, http://www.itksnap.org/pmwiki/pmwiki.php) which were used for manual segmentation. To correct for MRI intensity bias, a linear model was considered, e.g., *I*(*x*,*y*) = *g*(*x*,*y*)·*G*(*x*,*y*) + *b*(*x*,*y*) + *n*(*x*,*y*), where *g*(*x*,*y*) is the true signal intensity, *n*(*x*,*y*) represents the Gaussian random noise from background, *b*(*x*,*y*) is the additive interference representing the intensity bias independent of sample, and *G*(*x*,*y*) is the multiplicative interference. We then compared two other methods N4ITK and IM (Information Minimization) that were effective at lower field strength and found that both methods could not perform proper bias correction for our 9.4 T datasets. Therefore, an in-house developed P2 method was used to correct *b* (*x*, *y*), using a two-dimensional quadratic polynomial fitting algorithm for *b*(*x*, *y*). The fitted background bias is subtracted from the raw data, and the rest is the result *I*′(*x*, *y*) of the initial intensity bias correction. The multiplicative interference *G* (*x*, *y*) was determined to be proportional to the local additive noise *b* (*x*, *y*), e.g., *G*(*x*,*y*)∝*b*(*x*,*y*). Thus *g*(*x*, *y*) could be derived directly using *I*′ (*x*,*y*) / *b*(*x*,*y*). All data were normalized to [0 1] throughout the data processing.

### Antibodies and chemical reagents

Antibodies against OLIG2 (MABN50) and MRF (ABN45) were purchased from Millipore Abcam. Antibody against MBP (SMI-99) was purchased from Covance. Antibodies against NG2 (ab129051), GAFP (ab7260), and IBA1 (ab15060) were purchased from Abcam. Secondary antibodies used for immunocytochemistry were as follows: Alexa Fluor 488 goat anti-Rabbit (A11034), Alexa Fluor 594 donkey anti-mouse (A21203), and Alexa Fluor 594 chicken anti-Mouse (A21200) (Invitrogen), HRP-Goat Anti-Mouse IgG (H + L; SA00001-1, Proteintech), HRP-Goat Anti-Rabbit IgG (H + L; SA00001-2, Proteintech), all used at a dilution of 1:500. DAPI (4′,6′-diamidino-2-phenylindole; Invitrogen) was used as a nuclear counterstain at 1 µg/ml.

### Black-Gold II myelin stain

Visualization of fine intracortical myelin fibers in frozen sections was achieved by staining with Black-Gold II compound according to the manufacturer's instructions; cell nuclei were counterstained with cresyl violet (0.2% w/v; HistoChem). Briefly, after the macaque was perfused, the brain was fixed in 4% PFA at 4°C for 72 h, the right frontal lobe was removed, and then 20, 30, and 40% sucrose solutions were put into the gradient precipitation dehydration. Preparations before staining: equilibrate brain slices at room temperature for 20 min, preheat 0.3% Black-Gold II and 1% sodium thiosulfate solution at 60°C. Ultrapure water infiltration: the tissue is delineated by a histochemical pen, and the tissue section is soaked in ultrapure water for 2 min. Black-Gold II solution: add preheated Black-Gold II solution, incubate at 60°C for 12 min, and observe the tissue section under the microscope every 2–3 min until the thin parallel fibers of the first layer of the cortex are observed. When the finest myelinated fiber is dyed deep red to black, the dyeing is complete; rinse with ultrapure water: after dyeing is completed, rinse the sections with ultrapure water twice, each for 2 min; 1% sodium thiosulfate: drop 1% sodium thiosulfate, incubate at 60°C for 3 min; rinse with ultrapure water: rinse 3 times with ultrapure water, 2 min each time; gradient alcohol dehydration; xylene is transparent and can be sealed; observe under the microscope.

### Immunohistochemistry

For DAB/bright-field staining, 10 µm cryostat sections of the macaque brain were pretreated in 0.3% hydrogen peroxide in methanol for 30 min to remove endogenous peroxidase activity, rinsed in Tris-buffered saline (TBS), and then treated with 0.1 M citrate buffer in a microwave at sufficient power to keep the solution at 100°C for 20 min. Sections were cooled in the same buffer at room temperature (RT) for 30 min and rinsed in TBS. Slides were incubated in 10% goat serum in PBS blocking solution for 1 h at RT, after which primary antibodies MBP and Olig2 were applied to the sections that were then incubated at 4°C overnight. The sections were washed three times in TBS before applying the secondary antibody (Vector Laboratories). The secondary antibody was applied for 1 h at RT. Afterward, sections were rinsed three times in TBS. Rinsed sections were then incubated in Vectastain ABC Elite reagent for 1 h and developed using diaminobenzidine (Vector Laboratories). After dehydration, all sections were mounted in Permount under a glass coverslip. Control sections were subjected to the identical staining procedure, except for the omission of the primary antibody.

### Immunofluorescence

The rhesus macaque brain cryostat sections were first rinsed in PBS, followed by pretreatment in antigen unmasking solution (low pH) for 30 min at 100°C. After the slides had cooled in the buffer for 30 min at room temperature, slides were rinsed in PBS. Sections were incubated in 10% goat serum in PBS to block nonspecific binding for 1 h at room temperature, followed by incubation with the primary antibody against OLIG2 (MABN50, Millipore), MBP (SMI-99, Covance), and IBA1 (ab15060, Abcam) overnight at 4°C. Primary antibodies were visualized with the appropriate Alexa Fluor 488 and Alexa Fluor 594 secondary antibodies (Invitrogen). All sections were mounted in ProLong Gold antifade reagent with 4′,6-diamidino-2-phenylindole (DAPI; Invitrogen) under a glass coverslip. All immunofluorescence images were collected using a Zeiss Olympus IX-81 microscope with either a 40× or 100× objective running Metamorph. Microsoft Excel was used to calculate the fraction of positive clusters. GraphPad Prism was used to perform *t* tests and to visualize bar charts. Error bars represent SD.

### RNAscope assay for mRNA detection

Detection of macaque *Pdgfra* and *Myrf* were performed on cryostat brain sections of PFC of rhesus macaques using RNAscope Probes (*Pdgfra* XM-015138691 and *Myrf* NM-001194718, ACD) and RNAscope 2.5 LS Multiplex Fluorescent Reagent Kit v2 ASSAY (323100-USM, ACD). Positive (RNAscope 3-plex LS Multiplex Control Positive Probe, Mm polr2A, PPIB, ubc; ACD) and negative (RNAscope 3-plex LS Multiplex Negative Control Probe DAPB; ACD) controls were performed in parallel. Slides were thawed at room temperature for 10 min before baking at 60°C for 45 min. The sections were then postfixed in prechilled 4% PFA for 15 min at 4°C and washed in three changes of PBS for 5 min each before dehydration through 50, 70, 100, and 100% ethanol for 5 min each. The slides were air-dried for 5 min before loading onto a Bond Rx instrument (Leica Biosystems). Slides were prepared using the frozen slide delay before pretreatments using Epitope Retrieval Solution 2 (Leica Biosystems) at 95°C for 5 min and ACD Enzyme from the Multiplex Reagent kit at 40°C for 10 min. Probe hybridization and signal amplification were performed according to manufacturer's instructions. The following TSA plus fluorophores were used to detect corresponding RNAscope probes using the Bond Rx platform according to the ACD protocol: Fluorescein (Akoya Biosciences) and Cy5 (Akoya Biosciences) where slides were then removed from the Bond Rx and mounted using ProLong Diamond (Thermo Fisher Scientific). Slides were imaged on a CellDiscoverer 7 microscope (Zeiss). *Pdgfra*- and *Myrf*-positive cells were detected using the HALO FISH v2.1.6 analysis module based on intensity thresholds set using negative controls for both the fluorescein and Cy5 channels. Neurons detected as positive for *Pdgfra* and *Myrf* were checked by eye and were only included in the final analysis if there were three or more spots corresponding to *Pdgfra* and *Myrf* mRNAs. Probed regions of the target genes and sequences of target probes are seen in the product information of RNAscope probes (*Pdgfra* XM-015138691 and *Myrf* NM-001194718, ACD).

### Data correlation analysis

Data correlation analysis was conducted using Matlab 2021b, Excel 2019, and GraphPad Prism 9.2.

### Statistical analysis

All experimental data were analyzed statistically using GraphPad Prism (version 10.4.0) and Microsoft Office Excel (Microsoft 365). Experiments were conducted in triplicate or with three or more biological replicates, unless otherwise specified. Results are expressed as mean ± standard error of the mean (SEM). Statistical comparisons were performed using two-tailed Student's *t* tests or nonparametric Mann–Whitney *U* tests, as detailed in the figure legends. Assumptions of the statistical tests, including normality and equal variance, were verified. A two-sided paired *t* test was used to analyze differences in brain volume changes in macaques. Statistical significance for Black-Gold staining, immunohistochemistry, and RNAscope in situ hybridization were assessed using either unpaired *t* tests or one-way ANOVA with multiple comparisons, following normality testing with the Lilliefors test. Sample sizes were not predetermined using statistical methods but were consistent with standards commonly used in the field. Data collection and analysis were not blinded, and randomization was not applied. *p* values were calculated using a two-sided Wilcoxon rank-sum test or other statistical methods as specified in the figure legends and Materials and Methods section. Correlation analyses were performed using Spearman's correlation. A *p* value of <0.05 was considered statistically significant, with exact *p* values reported in the respective figures.

## Results

### MRI reveals age-related changes in neural fiber volume and density in rhesus macaques during postnatal development and aging

**Figure 1.**
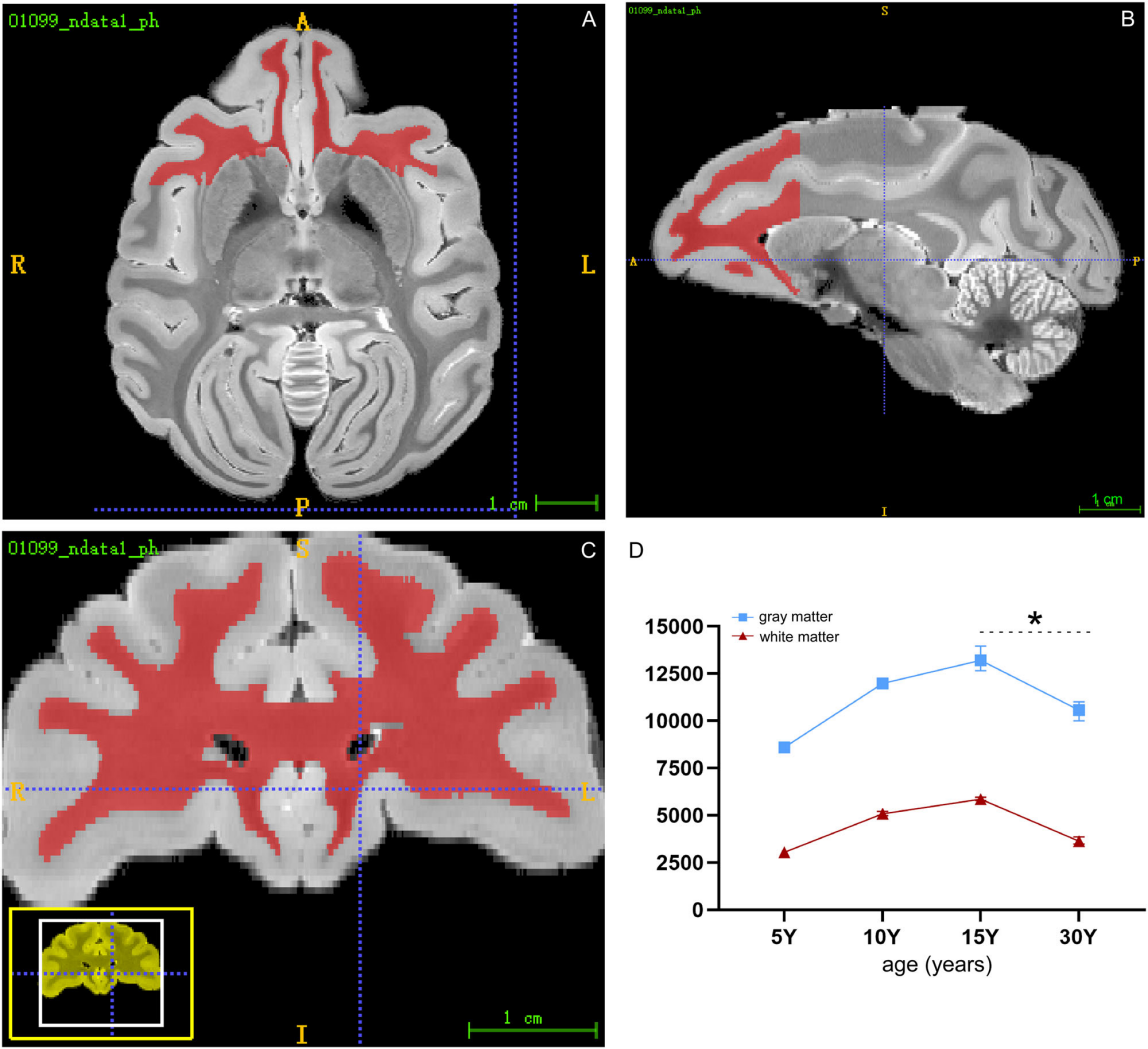
Age-related changes in white matter (red) and gray matter volume of the macaque brain. ***A–C***, Representative images of 15-year-old macaque brains in the horizontal, coronal, and sagittal planes using 9.4 T MRI. Scale bar, 1 cm. ***D***, Statistical analysis shows age-related changes in the volume of white matter (WM) and gray matter (GM) in the prefrontal lobe of macaques at 5, 10, 15, and 30 years of age (*n* = 5 per group, ANOVA, *p = 0.0036; ***p = 0.00078). Note: Detailed information about the macaques is provided in Extended Data Figure 1-1.

Rhesus macaques, as a primate species, share striking similarities with humans in terms of brain complexity, behavior, and cognitive abilities, making them invaluable models for studying age-associated brain changes. Their close genetic relationship with humans, supported by genomic and epigenomic studies, further underscores the potential for rhesus macaques to exhibit age-associated neural changes akin to those observed in humans, including demyelination and other brain alterations. In a previous study (Chen et al., 2020), we utilized a 9.4 T MRI imaging system to investigate structural changes in the brains of rhesus macaques throughout postnatal development and aging. This comprehensive analysis revealed notable reductions in both gray matter (GM) and white matter (WM) volumes, particularly in the prefrontal cortex, with increasing age (Fig. 1*A*–*D*). These findings describe age-associated volumetric changes in these regions. To facilitate a deeper understanding of the relationship between brain structure and aging, all the macaque subjects included in the study were summarized in Extended Data Figure 1-1, providing detailed information on their birth dates and scanning dates. Age was a key variable in this analysis, enabling the identification of meaningful correlations between changes in brain structure and the progression of aging.

10.1523/ENEURO.0418-25.2026.f1-1 Figure 1-1 Demographics and healthy state (HS) of rhesus macaques. Download Figure 1-1, DOCX file.

The MRI data were collected using various scanning sequences, which were segmented and analyzed to describe age-associated structural differences. Special attention was given to optimizing the scanning parameters, particularly the diffusion sensitivity (*b* value) in diffusion tensor imaging (DTI), to enhance the accuracy of the measurements. This methodological refinement allowed for a more detailed assessment of neural fiber volume and density changes, contributing to a comprehensive understanding of the neurobiological processes underlying aging in rhesus macaques.

### Age-related changes in myelin integrity in rhesus macaque brains during postnatal development and aging

**Figure 2.**
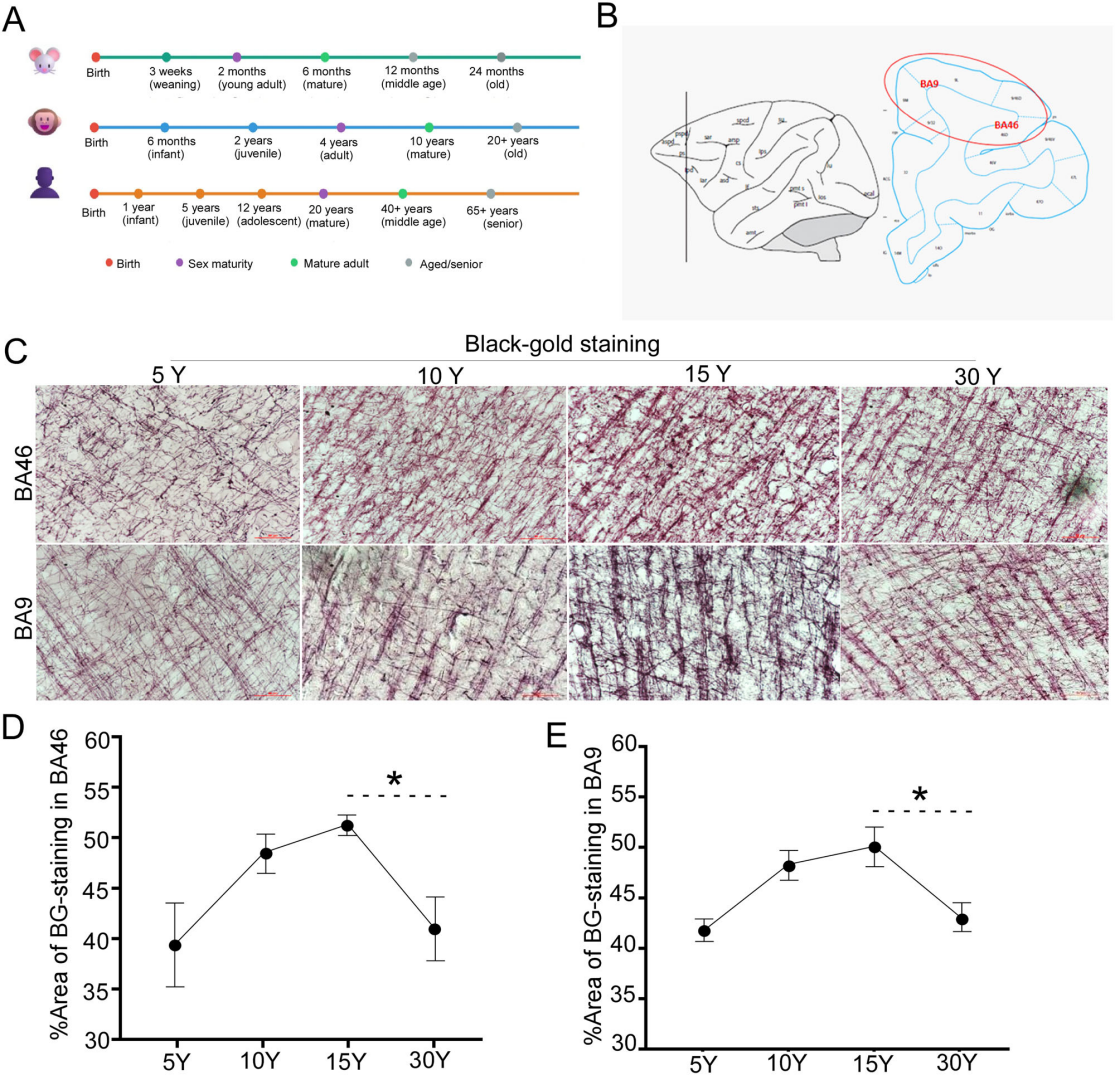
Age-related changes in myelin integrity in the macaque brain. ***A***, Schematic comparative age timeline across macaque, mouse, and human. ***B***, Schematic diagram of comparative Brodmann areas BA46 and BA9 in the prefrontal lobe of macaques. ***C***, Representative images of Black-Gold II staining demonstrating age-related changes in myelin density in BA46 and BA9 of the prefrontal lobe of macaques at ages 5, 10, 15, and 30 years. Scale bar, 200 µm. ***D***, ***E***, Relative intensities of Black-Gold II staining signals of myelin in panel ***B*** were quantified using ImageJ. Data are presented as mean ± SD (*n* = 5 macaques per age group; *p = 0.0026, unpaired *t* test). Note: Detailed information on brain myelin density for each macaque is provided in Extended Data Figures 2-1–2-4.

To contextualize our findings across model organisms, we established approximate age equivalencies between macaques, humans, and mice based on key developmental milestones (Fig. 2*A*). The timeline illustrates the compressed lifespan and accelerated development in rodents compared with primates. Previous studies have demonstrated that brain regions with delayed myelination maturity are more vulnerable to age-associated changes and other risk factors (Torkildsen et al., 2009). To investigate this hypothesis, we examined myelin content and density in the brains of rhesus macaques across postnatal development and aging. Using Black-Gold II staining, which selectively highlights myelinated fibers, we consistently visualized and quantified myelin-associated features in macaque brains at 5, 10, 15, and 30 years of age. Detailed information on brain myelin density for each macaque is provided in Extended Data Figures 2-1–2-4*.* The study focused on the dorsolateral prefrontal cortex, specifically Brodmann areas BA46 (upper lateral frontal cortex) and BA9 (posterolateral prefrontal cortex), which are closely associated with higher-order cognitive functions, including learning, working memory, and decision-making (Fig. 2*B*). These areas were selected due to their critical roles in cognition and their protracted developmental trajectories, making them particularly susceptible to aging-related alterations. Our observations indicate that myelin-associated measures in the prefrontal cortex undergo dynamic, age-associated changes (Fig. 2*C*–*E*). During early postnatal development, myelin density in the cortex steadily increases, consistent with ongoing myelination. This trend continues through adolescence and early adulthood, reaching a peak at ∼15 years of age. Myelin-associated measures appear maximally at this stage, consistent with the expected progression of cortical myelination. Beyond 15 years of age, a decline in both myelin content and density becomes evident, with significant reductions observed by 30 years of age.

10.1523/ENEURO.0418-25.2026.f2-1 Figure 2-1 Percentage of myelin in BA9 and BA49 in PFC of 5-year-old macaques. Download Figure 2-1, DOCX file.

10.1523/ENEURO.0418-25.2026.f2-2 Figure 2-2 Percentage of myelin in BA9 and BA49 in PFC of 10-year-old macaques. Download Figure 2-2, DOCX file.

**Figure 3.**
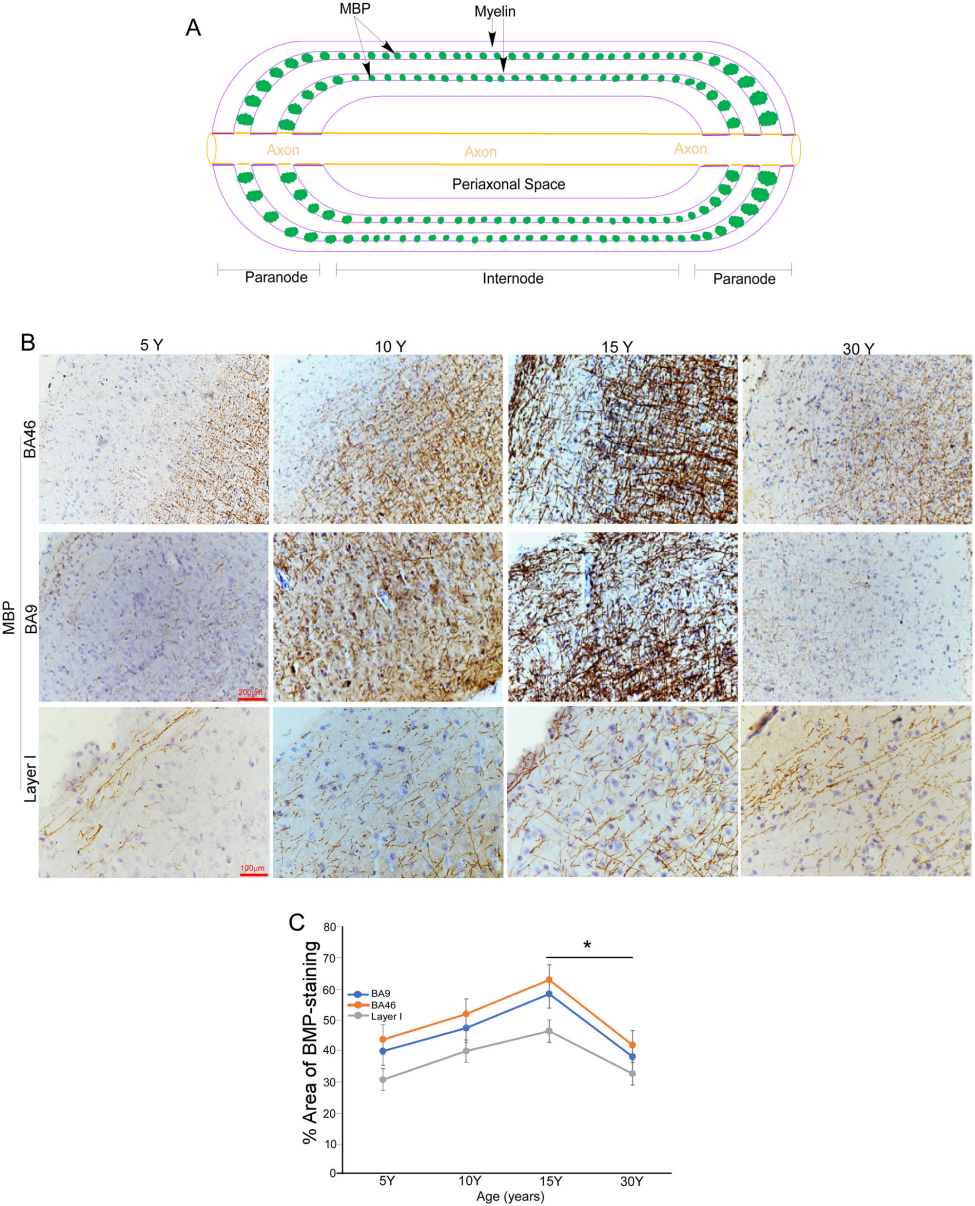
Age-related changes in the intensity of MBP signals in the macaque brain. ***A***, Schematic diagram illustrates the role of MBP in maintaining myelin sheath integrity. ***B***, Representative images of immunohistochemistry (IHC) showing MBP signals (brown) in Brodmann areas BA46 and BA9, as well as layer 1 of the prefrontal cortex (PFC) in macaques at ages 5, 10, 15, and 30 years. Scale bar as indicated. ***C***, Relative intensities of IHC MBP signals shown in panel ***B*** were quantified using ImageJ. Data are presented as mean ± SD (*n* = 5 macaques per age group; *p = 0.0038, unpaired *t* test).

10.1523/ENEURO.0418-25.2026.f2-3 Figure 2-3 Percentage of myelin in BA9 and BA49 in PFC of 15-year-old macaques. Download Figure 2-3, DOCX file.

**Figure 4.**
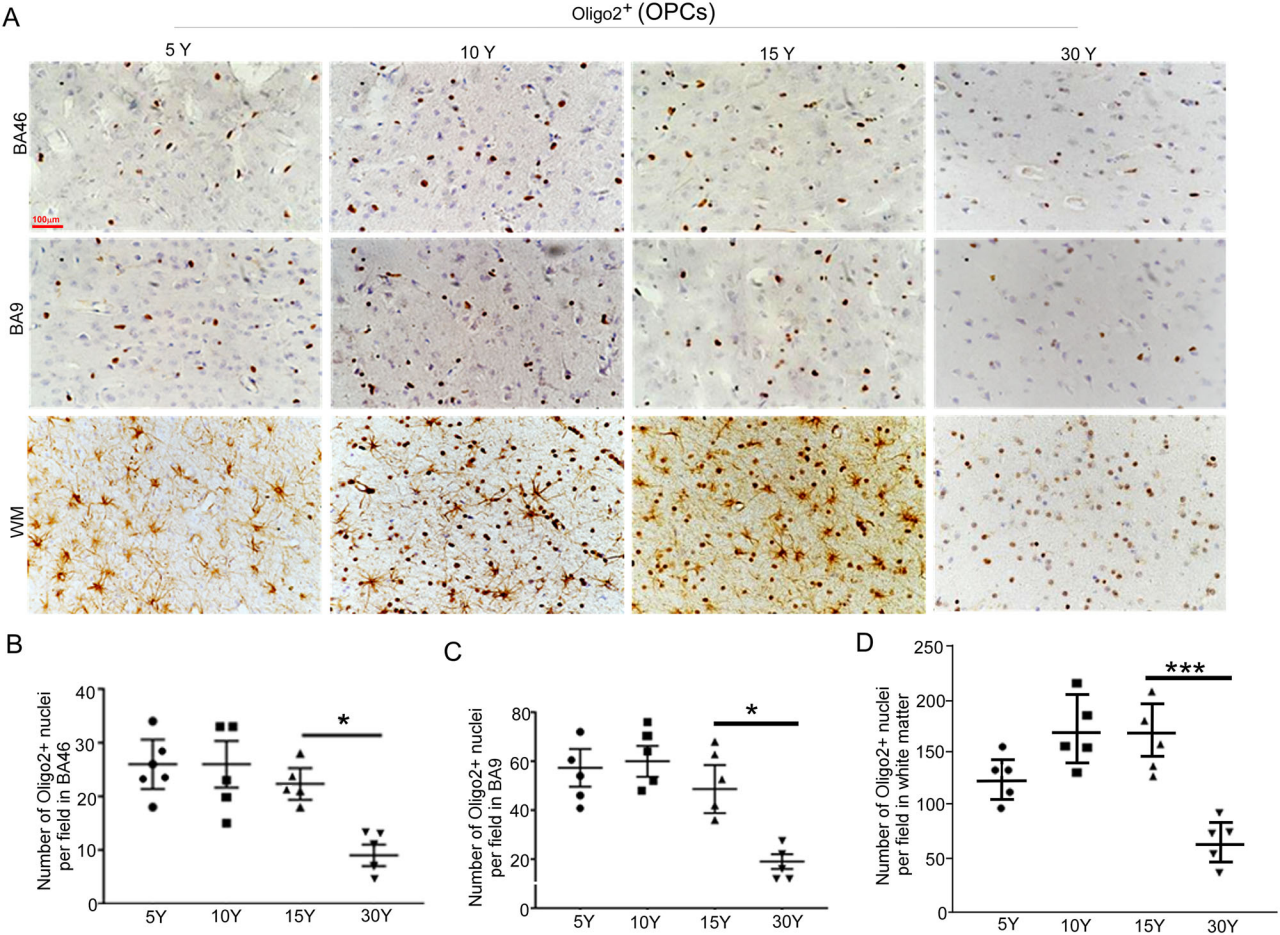
Age-related changes in the number of oligodendrocytes in the macaque brain. ***A***, Representative images of immunohistochemistry (IHC) showing Olig2-positive cells in the gray of Brodmann area BA46 and BA9 and white matter of PFC of macaques at ages 5, 10, 15, and 30 years. Scale bar, 100 µm. ***B–D***, Relative intensities of Olig2 signals shown in panel ***A*** were quantified using ImageJ. Data are presented as mean ± SD (*n* = 5 macaques per age group; *p = 0.0266; ***p = 0.00039, unpaired *t* test). Scale bar, 0.5 mm.

10.1523/ENEURO.0418-25.2026.f2-4 Figure 2-4 Percentage of myelin in BA9 and BA49 in PFC of 30-year-old macaques. Download Figure 2-4, DOCX file.

### Age-related changes in the intensities of MBP in rhesus macaque brains during postnatal development and aging

Myelin is a densely packed structure formed by multilayered membranes of oligodendrocytes that insulate axons. Myelin basic protein (MBP), a key marker of mature oligodendrocytes, plays an essential role in maintaining myelin integrity by acting as the “glue” that binds the layers of myelin membranes together. To investigate the contribution of oligodendrocytes to age-related declines in myelin integrity, we examined MBP expression in Brodmann areas BA46 (upper lateral frontal cortex) and BA9 (posterolateral prefrontal cortex) of rhesus macaque brains at 5, 10, 15, and 30 years of age (Fig. 3*A*–*C*). Our analysis revealed dynamic, age-associated changes in MBP signal intensity in the prefrontal cortex (PFC), generally following trends observed with Black-Gold II staining. Specifically, MBP expressions increased steadily during postnatal development, reaching a peak at ∼15 years of age. These observations describe age-associated increases in MBP signal, consistent with ongoing myelination during development. After 15 years, MBP intensity declined gradually, with lower signal observed by 30 years of age. We observed similar age-associated trends in MBP signal within layer 1 of the PFC, suggesting that changes in MBP signal occur across multiple cortical layers (Fig. 3*A*–*C*). The correspondence between MBP immunoreactivity and Black-Gold II staining provides complementary descriptive measures of myelin-associated features across age.

### Age-related changes in the number of Olig2-positive cells in rhesus monkey brains during postnatal development and aging

Myelin formation involves the proliferation, differentiation, and maturation of oligodendrocyte lineage cells (Lu et al., 2002; Zhang et al., 2019). To characterize age-associated changes in oligodendrocyte lineage cells, we quantified the number of Olig2-positive cells in the prefrontal cortex (PFC) of rhesus macaques at 5, 10, 15, and 30 years of age. Olig2 marks oligodendrocyte lineage cells, including both precursor and mature oligodendrocytes, but does not indicate functional state. Our analysis revealed age-associated patterns in Olig2-positive cell density in gray and white matter regions of the PFC. In the gray matter of BA46 and BA9, Olig2-positive cell numbers remained relatively stable up to 15 years of age (Fig. 4*A*–*C*) and declined by 30 years. These observations describe late-age reductions in Olig2-positive cell density in gray matter.

In contrast, a more pronounced decline in Olig2-positive cells was observed in white matter of the PFC by 30 years (Fig. 4*A*,*D*). These data describe age-associated decreases in Olig2-positive cell density in white matter relative to earlier ages and indicate that gray and white matter Olig2-positive cell densities exhibit different age-associated trajectories.

### Aging leads to decreases in the number of OPCs in rhesus macaque brains

**Figure 5.**
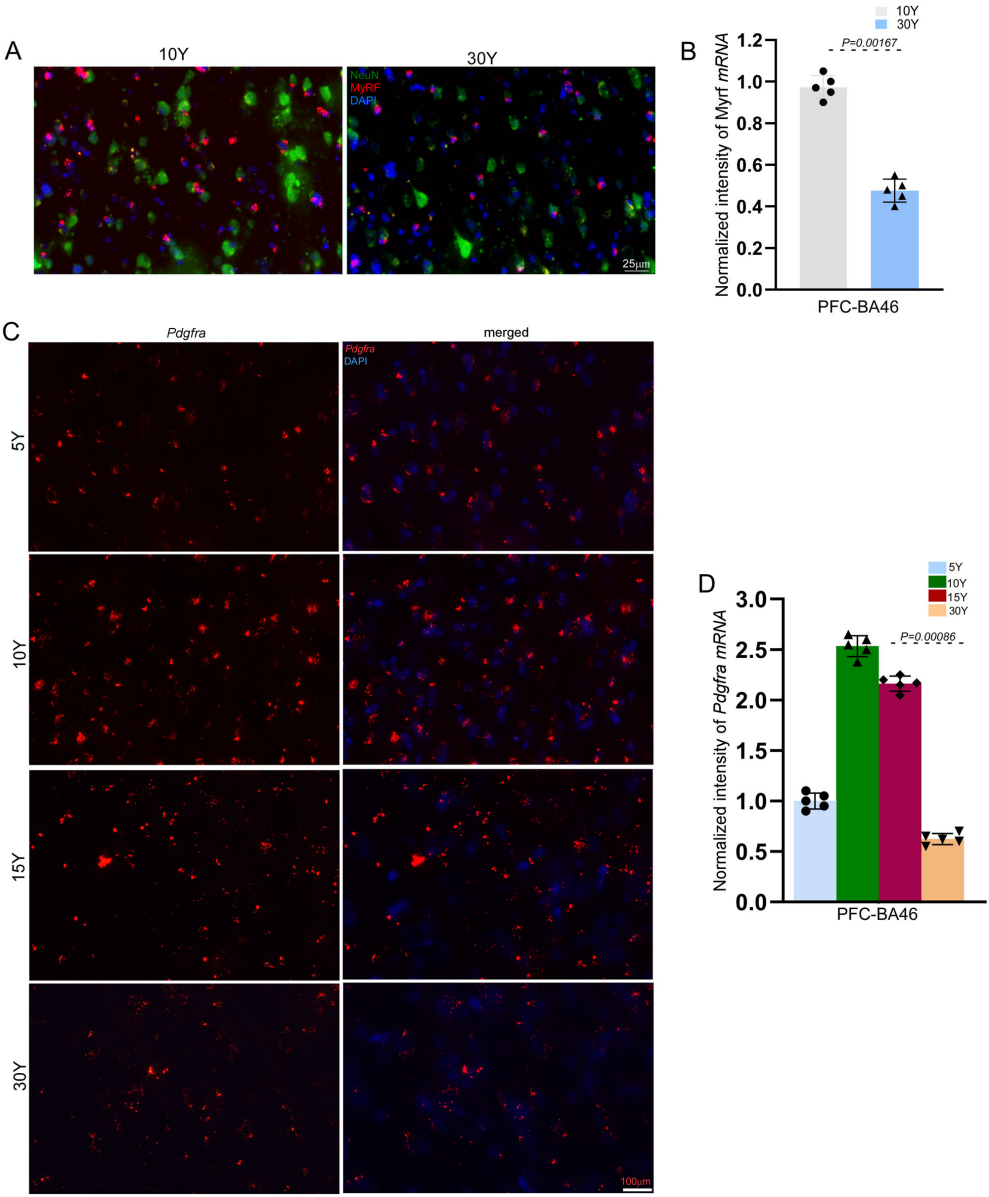
Aging leads to decreases in the number of matured oligodendrocytes and OPCs in the macaque brain. ***A***, Representative images of RNAscope in situ hybridization (ISH) showing Myrf-positive matured oligodendrocytes (red) in Brodmann area BA46 of the prefrontal cortex of macaques at ages 10 and 30 years. Immunofluorescence images of NEUN (green) indicate neuronal control. Scale bar, 25 µm. ***B***, Relative intensities of RNAscope ISH signals shown in panel ***A*** were quantified using ImageJ. Data are presented as mean ± SD (*n* = 5 macaques per age group; *p < 0.005, unpaired *t* test). ***C***, Representative images of RNAscope ISH showing Pdgfra-positive OPCs (red) in Brodmann area BA46 of the prefrontal cortex of macaques at ages 5, 10, 15, and 30 years. Scale bar, 100 µm. ***D***, Relative intensities of RNAscope ISH signals shown in panel ***C*** were quantified using ImageJ. Data are presented as mean ± SD (*n* = 5 macaques per age group; *p < 0.0001, unpaired *t* test).

Age-related declines in myelin integrity are closely tied to changes in the oligodendrocyte lineage, including oligodendrocyte precursor cells (OPCs) and mature oligodendrocytes. To describe age-associated changes in mature oligodendrocytes, we performed RNAscope in situ hybridization (ISH) to detect Myrf mRNA, which is expressed in oligodendrocyte lineage cells, including newly differentiated oligodendrocytes. Analysis of Myrf-positive cells in the prefrontal cortex (PFC), specifically BA46, revealed age-associated reductions in cell density in both white and gray matter in 30-year-old macaques compared with younger groups (Fig. 5*A*,*B*). To further characterize the relationship between age-related loss of MBP and Myrf-positive mature oligodendrocytes and broader changes in the oligodendrocyte lineage, we examined OPCs using platelet-derived growth factor receptor alpha (PDGFRα), a specific marker of OPCs. RNAscope ISH was employed to detect Pdgfra mRNA in BA46 of the PFC across macaques aged 5, 10, 15, and 30 years. Results showed that the number of Pdgfra-positive OPCs increased significantly from 5 to 10 years of age, coinciding with the critical period of dynamic myelination during postnatal brain development. However, a gradual decline in OPC numbers was observed from 15 years onward, culminating in a significant loss of OPCs in 30-year-old macaques (Fig. 5*C*,*D*). These observations describe age-associated increases in OPC density during early postnatal development and subsequent declines during aging. Overall, these data provide a descriptive account of oligodendrocyte lineage dynamics in the PFC across the lifespan of rhesus macaques.

### Accumulation of myelin debris in microglia is a hallmark of brain aging

**Figure 6.**
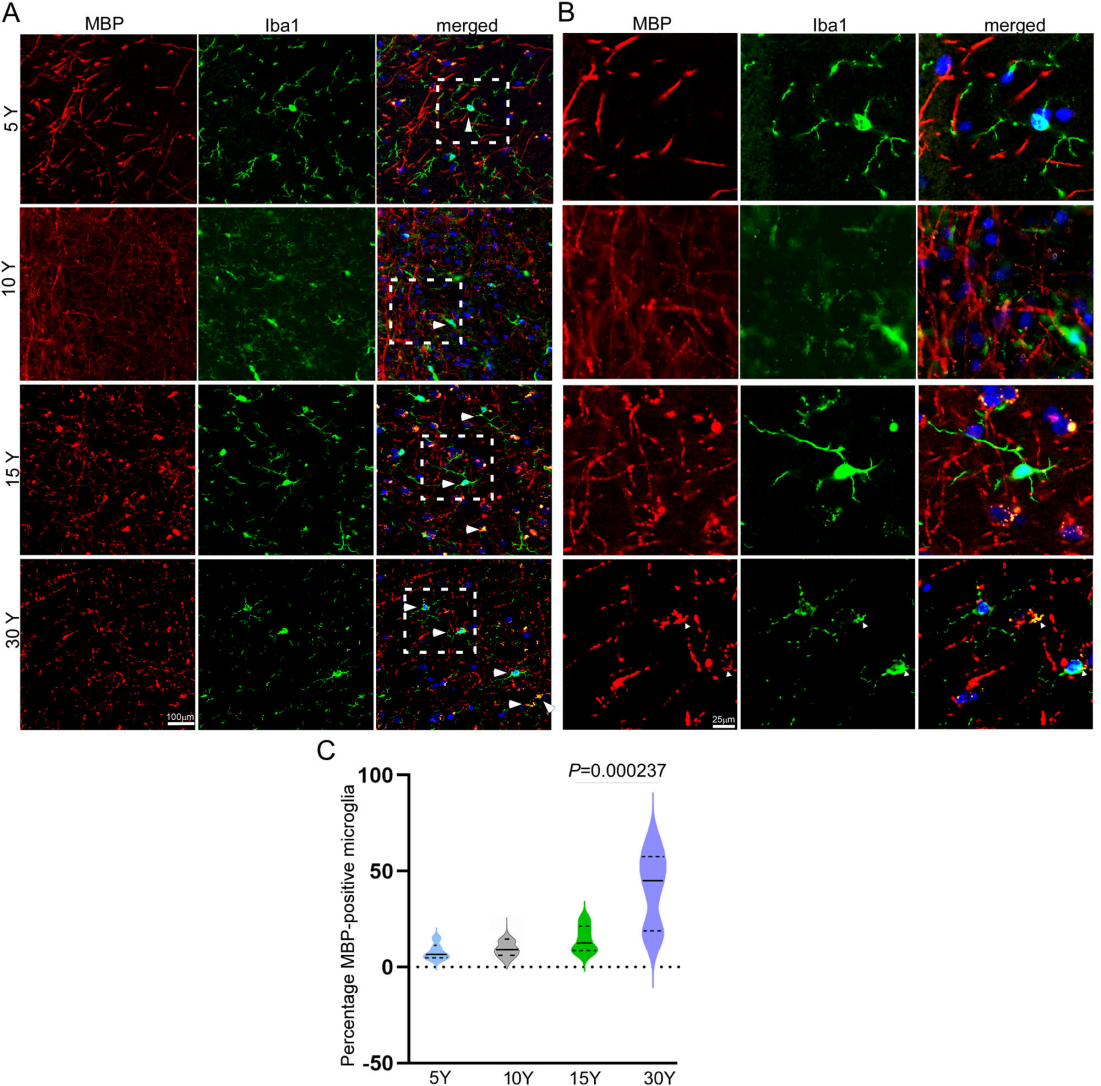
Accumulation of myelin debris in microglia in the aging macaque brain. ***A***, ***B***, Representative images of immunofluorescence staining showing microglia (green, Iba1) and myelin debris (red, MBP) in BA46 of the prefrontal cortex of macaques at the ages of 5, 10, 15, and 30 years. ***C***, Mean fold change in the number of myelin debris-accumulated microglia in BA46 of the macaque brain is represented by the horizontal line and is plotted for MBP (red) and Iba1 (green; *n* = 5 macaques per age group). Each symbol represents an individual macaque, with alterations referenced to the mean of the 5-year-old group. One-way ANOVA analysis showed a significant increase in the accumulation of myelin debris in microglia with age (*F*_(6,20)_ = 16.78, *p* < 0.0001), but no significant change was observed in the microglia marker Iba1.

Microglia, the brain's resident macrophages, are key mediators of immune surveillance and homeostasis within the central nervous system (CNS). They are known to participate in myelin clearance during brain development and in pathological conditions such as multiple sclerosis (MS; Kreutzberg, 1996). To describe age-associated changes in microglial association with myelin, we performed immunofluorescence costaining of ionized calcium-binding adapter molecule 1 (Iba1), a microglial marker, and MBP, a structural component of myelin, in BA46 of the prefrontal cortex (PFC) in rhesus macaques aged 5, 10, 15, and 30 years. Our analysis revealed age-associated increases in colocalization of Iba1 and MBP in 30-year-old macaques compared with younger groups (Fig. 6*A*–*C*). These observations describe increased association of microglial markers with myelin-associated signals in aged animals; no functional conclusions regarding microglial activity are drawn. These data indicate age-associated increases in microglia-associated myelin signal in BA46. The observations are consistent with previous reports of age-associated microglial changes.

## Discussion

Aging and aging-related neurodegenerative diseases remain incompletely understood, particularly with respect to how myelin-associated features and oligodendrocyte lineage cell populations change across the lifespan. Neuropathological studies have highlighted the relationship between age-related changes in myelin sheaths, oligodendrocytes, and overall brain aging (Liu et al., 2012; Zatorre et al., 2012; Fields, 2015). However, the temporal associations between myelin integrity changes, oligodendrocyte dynamics, and their precursor cells during aging, especially in the primate brain, remain poorly defined. Furthermore, the spatiotemporal evolution of these processes in the structurally complex primate brain remains elusive. This study addresses these gaps by providing a descriptive, cross-sectional dataset of age-associated changes in myelin-related imaging measures and oligodendrocyte lineage-associated markers in a translationally relevant primate model.

Research in rodent models has established that cortical myelination plays a role in learning, memory, cognition, and neural circuit plasticity (Fields, 2015). Nevertheless, there has been limited focus on gray matter myelination and its changes during aging, particularly in primates. Rhesus macaques, as close relatives of humans, offer a valuable model due to their physiological and structural similarities to the human brain. By leveraging high-resolution 9.4 T MRI, histological analyses, and molecular assessments, we examined age-associated changes in myelin-related measures and oligodendrocyte lineage markers across four distinct age groups (5, 10, 15, and 30 years). Our MRI analyses revealed age-related reductions in the volume of both gray and white matter in the prefrontal cortex (PFC), consistent with physiological brain atrophy observed in aging primates and humans. Black-Gold II staining demonstrated that myelin density peaks at ∼15 years of age, followed by an age-related decline. While Black-Gold II staining provides a measure of myelin content rather than direct ultrastructural integrity, this observation, when combined with our volumetric MRI data, indicates that age-associated changes in myelin content occur alongside reductions in PFC volume during aging. Nevertheless, the biological processes underlying these changes remain to be determined. We acknowledge that multiple factors beyond oligodendrocyte dysfunction, including oxidative stress, mitochondrial dysfunction, vascular alterations, chronic inflammatory processes, and metabolic dysregulation, may contribute to myelin deterioration during aging. Whether these factors act independently or synergistically to drive myelin loss warrants investigation in future mechanistic studies.

At the cellular level, the oligodendrocyte lineage was analyzed to characterize the temporal dynamics of myelin-associated cells during aging. Olig2-positive cells, which encompass the entire oligodendrocyte lineage including both OPCs and mature oligodendrocytes, exhibited sustained numbers and were not significantly reduced until 30 years of age. Of note, Olig2 expression alone does not distinguish between OPC and mature oligodendrocyte populations, nor does it provide information about cellular functional states. In contrast, examination of MBP-positive cells revealed a pronounced decline with aging, particularly in layer 1 of the PFC. This decrease in MBP immunoreactivity temporally coincides with reductions in myelin-associated imaging measures. Indeed, reduced MBP intensity may result from multiple factors, including decreased expression per cell, reduced oligodendrocyte numbers, or alterations in myelin structure, and our data do not distinguish among these possibilities. Further analysis using RNAscope revealed significant age-related reductions in OPCs (Pdgfra-positive cells) and Myrf-positive cells. These findings indicate age-associated changes in the density of cells expressing OPC- and oligodendrocyte lineage-associated transcripts. The age-associated changes in OPC density temporally coincide with alterations in myelin-related measures during aging. However, we cannot infer causal relationships from these descriptive data. It is equally plausible that primary myelin damage, potentially driven by metabolic, inflammatory, or vascular factors, occurs first and subsequently impacts OPC survival and differentiation. Distinguishing between these scenarios would require mechanistic interventions, such as experimental enhancement of OPC survival or proliferation, to determine whether such manipulations can prevent myelin loss.

Another notable observation in this study was the accumulation of myelin debris in microglia. Immunofluorescence costaining of Iba1 and MBP revealed increased colocalization in 30-year-old macaques compared with younger animals, suggesting increased myelin debris within or associated with microglia during aging. While this pattern may reflect altered myelin turnover or clearance, our data do not directly assess microglial phagocytic function or inflammatory status. Future studies incorporating functional phagocytosis assays and cytokine profiling would be necessary to determine whether aged microglia exhibit reduced clearance efficiency or altered inflammatory responses that contribute to myelin pathology. Our findings in BA9 and BA46 of the PFC provide detailed temporal characterization of myelin and oligodendrocyte changes in regions critical for executive function and working memory. However, we acknowledge that these observations may not generalize to all brain regions, as regional heterogeneity in myelination patterns and oligodendrocyte dynamics has been documented in both rodent and human studies. Whether the age-related patterns we observe represent region-specific phenomena or reflect broader brain-wide processes remain to be determined through examination of additional cortical and subcortical areas.

When comparing our findings to the existing literature, several consistencies and notable differences emerge. Like studies in rodent models, we observed age-related myelin decline and OPC depletion, suggesting conserved aspects of oligodendrocyte aging across mammalian species. However, the extended lifespan of primates allows examination of these processes over decades rather than months, potentially revealing temporal dynamics not observable in shorter-lived species. Our observations of peak myelination at 15 years followed by decline aligning with human neuroimaging studies showing white matter volume peaks in middle adulthood, though the correspondence between macaque and human aging trajectories requires careful consideration given species-specific developmental timelines. As there are differences from some rodent studies reporting sustained OPC proliferation and myelin repair capacity, our data indicate progressive OPC depletion in aged macaques, which may reflect fundamental differences in regenerative capacity between rodents and primates or could be influenced by the different methodological approaches employed. Together, these findings provide a descriptive framework for understanding age-associated changes in myelin-related features in the primate brain. The temporal correlations we identified between OPC loss, reduced mature oligodendrocyte markers, and myelin decline provide a foundation for future mechanistic investigations. Myelin-based markers, such as MBP and OPC-related proteins, may serve as accessible biomarkers for tracking brain aging in longitudinal studies. Integrating MRI imaging with cellular and molecular assessments of oligodendrocytes and myelin can provide multiscale perspectives on structural brain changes during aging.

While this study provides valuable insight into age-associated changes in myelin-related features in the primate brain, several limitations should be acknowledged. First, cross-sectional design limits inference about individual longitudinal trajectories of myelin-associated imaging measures and oligodendrocyte lineage marker expression across the lifespan. Second, although the rhesus macaque model closely parallels human brain aging, species-specific differences in myelin turnover and lifespan may influence the generalizability of our findings. Third, the analysis focused on prefrontal cortex regions BA9 and BA46; additional brain areas may exhibit distinct myelin aging patterns. Fourth, our sample size of five animals per age group, while typical for nonhuman primate studies, limits statistical power for detecting subtle effects and precludes sex-stratified analyses. Fifth, Black-Gold II staining provides measures of myelin content rather than direct ultrastructural integrity, and MBP intensity reductions may reflect multiple underlying factors. Finally, this study is descriptive in nature and does not address causal relationships between oligodendrocyte lineage changes and myelin-associated outcomes. Future studies would employ interventional approaches to test whether enhancing OPC survival or proliferation can mitigate myelin loss, thereby establishing causality. Direct assessment of microglial phagocytic capacity and inflammatory profiles would clarify their functional role in age-related myelin changes. Sex-specific analyses, examination of additional brain regions, and single-cell molecular profiling would provide comprehensive understanding of myelin aging mechanisms. Such mechanistic insights will inform the development of therapeutic strategies to promote healthy brain aging across the lifespan.

### Conclusion

Our findings demonstrate myelin integrity and oligodendrocyte lineage dynamics in the prefrontal cortex of rhesus macaque across development and aging, with maturation peaking in adolescence and declining in later life. The convergence of imaging and cellular markers highlights tightly linked structural and cellular mechanisms underlying age-related myelin remodeling. This lifespan framework provides an insight for investigating mechanisms of myelin vulnerability and resilience in aging and age-associated brain disorders.

## Synthesis

Reviewing Editor: Benjamin Deneen, Baylor College of Medicine Decisions are customarily a result of the Reviewing Editor and the peer reviewers coming together and discussing their recommendations until a consensus is reached. When revisions are invited, a fact-based synthesis statement explaining their decision and outlining what is needed to prepare a revision will be listed below. The following reviewer(s) agreed to reveal their identity: NONE. Note: If this manuscript was transferred from JNeurosci and a decision was made to accept the manuscript without peer review, a synthesis may not be available.

We had some difficulty attaining two reviewers, so we have one comprehensive review. I have also looked over the paper and agree with the comments from the reviewer. Please revise accordingly.

## Author Response

Response to reviewers' comments We sincerely thank the reviewer for their careful and constructive evaluation of our manuscript. We appreciate the reviewer's recognition of the potential value of our dataset for investigators studying oligodendrocyte lineage dynamics across the primate lifespan. We fully acknowledge the concerns regarding overinterpretation and imprecise language, and we have substantially revised the manuscript to improve accuracy, clarity, and consistency between the data presented and the conclusions drawn. Detailed point-by-point responses are provided below.

1. Overinterpretation in the title and abstract regarding OPC loss and mechanistic insight Reviewer comment:

The conclusion presented in the title and abstract is not supported by the data. OPC density changes do not demonstrate OPC loss, and no mechanistic insight is provided. Statements regarding decreased numbers of MBP-expressing mature oligodendrocytes are also inaccurate.

Response: We agree with the reviewer and thank them for highlighting these critical issues. In response, we revised the title to remove causal and mechanistic language and to more accurately reflect the descriptive scope of the study. The abstract has been rewritten to avoid claims of OPC "loss," mechanistic causality, or functional inference. We now explicitly describe age-associated changes in marker expression and density without attributing these changes to cell loss, impaired proliferation, or altered differentiation, and we have removed all statements suggesting that OPC alterations represent a "primary driver" of myelin degeneration. Additionally, we corrected the inaccurate statement regarding the "number of MBP-expressing mature oligodendrocytes." As correctly noted by the reviewer, MBP labels myelin sheaths rather than oligodendrocyte cell bodies. The revised text now accurately interprets MBP immunoreactivity as an index of myelin integrity rather than oligodendrocyte number.

2. Speculative and functional interpretations in the Results section Reviewer comment:

Statements suggesting functional roles of myelin (e.g., supporting neural network efficiency) are not supported by the data.

Response: Thank the reviewer for this valuable suggestion. The Results section has been carefully revised to remove speculative or functional interpretations that are not directly supported by our measurements. Statements such as "supporting neural network efficiency" have been deleted or relocated to the Discussion and appropriately qualified. The Results section now focuses exclusively on quantitative observations, including marker expression patterns and age- associated trends, without extrapolation to functional outcomes.

3. Interpretation of U-shaped OPC density as a 'dual role' and use of unclear terminology A U-shaped density distribution does not indicate a 'dual role' for OPCs. The term "primitive oligodendrocyte progenitor cells" is unclear and unsupported.

Response: We appreciate the referee's valuable comment. We have removed the phrase "dual role" and now describe the U-shaped pattern strictly as an age-associated change in OPC density, without implying functional or developmental roles. In addition, the term "primitive oligodendrocyte progenitor cells" has been removed, as it was imprecise and not supported by specific markers or references. The revised text now employs standard, well-defined terminology consistent with the oligodendrocyte lineage literature.

4. Missing references for statements referring to existing literature Reviewer comment:

Several statements appear to reference prior work but lack citations.

Response: We thank the reviewer for highlighting this point. Appropriate references have been added to support statements on myelin maturation, structural plasticity, cognitive development, microglial involvement, and lipid metabolism. Statements lacking direct support have been softened or removed to avoid overgeneralization.

5. Inaccurate interpretation of Olig2+ cell data and functional capacity Reviewer comment:

Olig2 is not specific to oligodendrocytes and does not provide information about functional capacity.

Response: We thank the reviewer for this valuable suggestion. All interpretations of Olig2⁺ cell density as reflecting oligodendrocyte function or vulnerability have been revised. The manuscript now clarifies that Olig2 labels multiple stages of the oligodendrocyte lineage (and some astrocytes) and that changes in Olig2⁺ density reflect lineage composition rather than functional competence. Any language suggesting functional decline has been removed.

6. Request for an age-benchmarking summary figure Reviewer comment:

A summary figure comparing macaque ages to approximate human and rodent ages would be helpful.

Response: We appreciate this helpful suggestion. A new schematic summary panel (now included as Figure 2A) has been added to illustrate the approximate age equivalence between macaques, humans, and rodents.

7. Use of Myrf as a proxy for mature oligodendrocytes Myrf is expressed across the oligodendrocyte lineage and should not be used as a proxy for mature oligodendrocyte number.

Response: Thank the reviewer for this helpful comment. Myrf expression is no longer interpreted as a direct measure of mature oligodendrocyte number. The text now clarifies that Myrf is expressed across multiple stages of the lineage and serves as an indicator of oligodendrocyte differentiation state rather than cell abundance, and conclusions based on Myrf data have been correspondingly tempered.

8. Response to Reviewer Comments on Study Scope and Interpretation:

Thank the reviewer for highlighting concerns regarding over-interpretation and causal language in the original manuscript. In response, we have revised the title, abstract, Discussion, and Limitations sections to emphasize the descriptive and observational nature of our study. The title and abstract no longer suggest that OPC loss drives myelin decline; instead, they now accurately describe age-associated changes in myelin-related markers and oligodendrocyte lineage cell density in the prefrontal cortex of rhesus macaques. In the Discussion, we have removed speculative functional claims, clarified the interpretation of Olig2, MBP, and Myrf markers, and focused on temporal and correlative patterns rather than mechanistic conclusions. The Limitations section has been rewritten to explicitly acknowledge that the study is cross-sectional, limited to BA9 and BA46, and that marker-based measures cannot establish causality. Together, these revisions ensure that the manuscript accurately reflects the scope of the data, aligns with the reviewer's suggestions, and provides a clear, rigorous reference framework for future mechanistic studies of myelin and oligodendrocyte lineage dynamics in primates.

9. Response regarding Limitations:

Thank the reviewer for highlighting the need to clarify the scope of our study. In response, we have substantially revised the Limitations section to remove causal or mechanistic language and to emphasize the descriptive nature of our findings. We now explicitly acknowledge that our study does not establish causal relationships between oligodendrocyte lineage changes and myelin-associated features. Additionally, we have clarified the constraints of our cross-sectional design, limited brain regions examined, sample size, and marker-specific interpretations. These revisions ensure the Limitations section accurately reflects the observational scope of the study and align with the revised framing of the title, abstract, and discussion.
