# Supplementary material for: Age-Related Decline in Myelin Markers and Oligodendrocyte Density in Rhesus Macaque Prefrontal Cortex
Source: eNeuro. 2026 Apr 14;13(4):ENEURO.0418-25.2026. doi: 10.1523/ENEURO.0418-25.2026 (PMC13102401; doi:10.1523/ENEURO.0418-25.2026)
Supplement: Figure 1-1 — Demographics and healthy state (HS) of rhesus macaques. Download Figure 1-1, DOCX file. [file eneuro-13-ENEURO.0418-25.2026-s002.docx]

**Figure 1-1** Demographics and healthy state (HS) of rhesus macaques

| No. | Age (years) Sex HS |
| --- | --- |
| 14397 | 5 F good |
| 14050 | 5 F good |
| 14076  14056  14082  09076 | 5 M good  5 F good  5 M good  10 M good |
| 09370 | 10 M good |
| 09352 | 10 F good |
| 09084 | 10 M good |
| 09026 | 10 F good |
| 04052 | 15 F good |
| 04009  04006  04452 | 15 M good  15 F good  15 F good |
| 04374 | 15 M good |
| 89330 | 30 M good |
| 89036 | 30 F good |
| 89309 | 30 M good |
| 89323 | 30 F good |
| 89313 | 30 M good |
