# Supplementary material for: Age-Related Decline in Myelin Markers and Oligodendrocyte Density in Rhesus Macaque Prefrontal Cortex
Source: eNeuro. 2026 Apr 14;13(4):ENEURO.0418-25.2026. doi: 10.1523/ENEURO.0418-25.2026 (PMC13102401; doi:10.1523/ENEURO.0418-25.2026)
Supplement: Figure 2-1 — Percentage of myelin in BA9 and BA49 in PFC of 5-year-old macaques. Download Figure 2-1, DOCX file. [file eneuro-13-ENEURO.0418-25.2026-s003.docx]

**Figure 2-1**. Percentage of myelin in BA9 and BA49 in PFC of 5-year-old macaques

| Group | No |  | BA9 | | BA46 | |
| --- | --- | --- | --- | --- | --- | --- |
|  |  |  | myelin（%） | average（%） | myelin（%） | average（%） |
| Y5 | 14397 |  | 39.923 | 40.094 | 41.296 | 40.091 |
|  |  |  | 41.447 |  | 39.851 |  |
|  |  |  | 38.911 |  | 39.128 |  |
|  | 14050 |  | 41.649 | 40.578 | 40.139 | 40.395 |
|  |  |  | 40.449 |  | 39.807 |  |
|  |  |  | 39.636 |  | 41.238 |  |
|  | 14076 |  | 39.822 | 40.295 | 40.617 | 40.620 |
|  |  |  | 40.868 |  | 40.641 |  |
|  |  |  | 40.198 |  | 40.603 |  |
|  | 14056 |  | 39.608 | 39.422 | 41.114 | 41.374 |
|  |  |  | 40.067 |  | 40.486 |  |
|  |  |  | 38.591 |  | 42.523 |  |
|  | 14082 |  | 40.148 | 39.598 | 40.869 | 40.602 |
|  |  |  | 39.287 |  | 40.074 |  |
|  |  |  | 39.369 |  | 40.679 |  |
