# Supplementary material for: Age-Related Decline in Myelin Markers and Oligodendrocyte Density in Rhesus Macaque Prefrontal Cortex
Source: eNeuro. 2026 Apr 14;13(4):ENEURO.0418-25.2026. doi: 10.1523/ENEURO.0418-25.2026 (PMC13102401; doi:10.1523/ENEURO.0418-25.2026)
Supplement: Figure 2-2 — Percentage of myelin in BA9 and BA49 in PFC of 10-year-old macaques. Download Figure 2-2, DOCX file. [file eneuro-13-ENEURO.0418-25.2026-s004.docx]

**Figure 2-2**. Percentage of myelin in BA9 and BA49 in PFC of 10-year-old macaques

| Group | No |  | BA9 | | BA46 | |
| --- | --- | --- | --- | --- | --- | --- |
|  |  |  | myelin（%） | average（%） | myelin（%） | average（%） |
| Y10 | 09076 |  | 42.893 | 43.102 | 42.287 | 42.088 |
|  |  |  | 42.451 |  | 42.861 |  |
|  |  |  | 43.906 |  | 41.132 |  |
|  | 09370 |  | 42.656 | 43.567 | 42.146 | 42.402 |
|  |  |  | 44.451 |  | 43.787 |  |
|  |  |  | 43.632 |  | 41.242 |  |
|  | 09352 |  | 43.831 | 43.326 | 43.599 | 43.631 |
|  |  |  | 42.798 |  | 43.636 |  |
|  |  |  | 43.201 |  | 43.612 |  |
|  | 09084 |  | 42.656 | 42.419 | 43.126 | 43.398 |
|  |  |  | 42.036 |  | 44.521 |  |
|  |  |  | 42.609 |  | 42.436 |  |
|  | 09026 |  | 42.143 | 42.623 | 43.865 | 44.468 |
|  |  |  | 41.268 |  | 43.163 |  |
|  |  |  | 44.382 |  | 46.576 |  |
