# Supplementary material for: Age-Related Decline in Myelin Markers and Oligodendrocyte Density in Rhesus Macaque Prefrontal Cortex
Source: eNeuro. 2026 Apr 14;13(4):ENEURO.0418-25.2026. doi: 10.1523/ENEURO.0418-25.2026 (PMC13102401; doi:10.1523/ENEURO.0418-25.2026)
Supplement: Figure 2-3 — Percentage of myelin in BA9 and BA49 in PFC of 15-year-old macaques. Download Figure 2-3, DOCX file. [file eneuro-13-ENEURO.0418-25.2026-s005.docx]

**Figure 2-3**. Percentage of myelin in BA9 and BA49 in PFC of 15-year-old macaques

| Group | No |  | BA9 | | BA46 | |
| --- | --- | --- | --- | --- | --- | --- |
|  |  |  | myelin（%） | average（%） | myelin（%） | average（%） |
| Y15 | 04052 |  | 46.786 | 46.135 | 46.265 | 45.106 |
|  |  |  | 44.563 |  | 44.823 |  |
|  |  |  | 45.889 |  | 45.213 |  |
|  | 04009 |  | 45.667 | 45.476 | 44.342 | 44.403 |
|  |  |  | 44.512 |  | 45.475 |  |
|  |  |  | 46.587 |  | 43.368 |  |
|  | 04006 |  | 44.913 | 44.187 | 44.548 | 45.326 |
|  |  |  | 43.867 |  | 45.377 |  |
|  |  |  | 45.073 |  | 46.078 |  |
|  | 04452 |  | 46.522 | 45.286 | 45.139 | 45.218 |
|  |  |  | 45.676 |  | 46.098 |  |
|  |  |  | 44.673 |  | 44.588 |  |
|  | 04374 |  | 46.426 | 46.063 | 45.893 | 45.543 |
|  |  |  | 45.603 |  | 44.963 |  |
|  |  |  | 46.176 |  | 45.689 |  |
