# Supplementary material for: Age-Related Decline in Myelin Markers and Oligodendrocyte Density in Rhesus Macaque Prefrontal Cortex
Source: eNeuro. 2026 Apr 14;13(4):ENEURO.0418-25.2026. doi: 10.1523/ENEURO.0418-25.2026 (PMC13102401; doi:10.1523/ENEURO.0418-25.2026)
Supplement: Figure 2-4 — Percentage of myelin in BA9 and BA49 in PFC of 30-year-old macaques. Download Figure 2-4, DOCX file. [file eneuro-13-ENEURO.0418-25.2026-s006.docx]

**Figure 2-4**. Percentage of myelin in BA9 and BA49 in PFC of 30-year-old macaques

| Group | No |  | BA9 | | BA46 | |
| --- | --- | --- | --- | --- | --- | --- |
|  |  |  | myelin（%） | average（%） | myelin（%） | average（%） |
| Y30 | 89330 |  | 35.684 | 33.641 | 34.464 | 35.691 |
|  |  |  | 35.221 |  | 34.126 |  |
|  |  |  | 30.018 |  | 38.483 |  |
|  | 89036 |  | 33.082 | 33.518 | 35.956 | 35.594 |
|  |  |  | 34.258 |  | 33.997 |  |
|  |  |  | 33.226 |  | 36.830 |  |
|  | 89309 |  | 30.798 | 31.644 | 34.142 | 34.355 |
|  |  |  | 34.098 |  | 33.567 |  |
|  |  |  | 30.045 |  | 35.356 |  |
|  | 89323 |  | 34.446 | 34.069 | 35.958 | 35.204 |
|  |  |  | 34.509 |  | 33.682 |  |
|  |  |  | 33.253 |  | 35.972 |  |
|  | 89313 |  | 34.129 | 32.602 | 33.877 | 34.543 |
|  |  |  | 31.297 |  | 33.063 |  |
|  |  |  | 32.379 |  | 36.736 |  |

Notes: Each group includes 5 samples, and each sample includes 3 slices of BA46 and BA9. The area proportion of myelin sheath was calculated using Image J.
